# Agarase: Review of Major Sources, Categories, Purification Method, Enzyme Characteristics and Applications

**DOI:** 10.3390/md8010200

**Published:** 2010-01-26

**Authors:** Xiao Ting Fu, Sang Moo Kim

**Affiliations:** 1 College of Food Science and Engineering, Ocean University of China, Qingdao 266003, China; E-Mail: xiaotingfu@ouc.edu.cn; 2 Faculty of Marine Bioscience and Technology, Gangneung-Wonju National University, Gangneung 210-702, Korea

**Keywords:** agar, agarase, GH-16 family, GH-50 family, oligosaccharides

## Abstract

Agarases are the enzymes which catalyze the hydrolysis of agar. They are classified into α-agarase (E.C. 3.2.1.158) and β-agarase (E.C. 3.2.1.81) according to the cleavage pattern. Several agarases have been isolated from different genera of bacteria found in seawater and marine sediments, as well as engineered microorganisms. Agarases have wide applications in food industry, cosmetics, and medical fields because they produce oligosaccharides with remarkable activities. They are also used as a tool enzyme for biological, physiological, and cytological studies. The paper reviews the category, source, purification method, major characteristics, and application fields of these native and gene cloned agarases in the past, present, and future.

## 1. Introduction

Agarases catalyze the hydrolysis of agar. They are classified into α-agarase (E.C. 3.2.1.158) and β-agarase (E.C. 3.2.1.81) according to the cleavage pattern. The basic structure of agar is composed of repetitive units of β-d-galactose and 3,6-anhydro-α-l-galactose [1]. α-Agarases cleave α-1,3 linkages to produce agarooligosaccharides of series related to agarobiose [2], while β-agarases cleave β-1,4 linkages to produce neoagarooligosaccharides of series related to neoagarobiose [3]. So far, several agarases have been isolated from different genera of bacteria found in seawater, marine sediments and other environments. Agarases have a wide variety of applications. They have been used to hydrolyze agar to produce oligosaccharides, which exhibit important physiological and biological activities beneficial to the health of human being [4]. Besides that, agarases also have other uses as tools to isolate protoplasts from seaweeds [5] and to recover DNA from agarose gel [6], and to investigate the composition and structure of cell wall polysaccharide of seaweeds. Recent progress in cloning and sequencing of these enzymes has led to structure-function analyses of agarase [7–10]. This information will provide valuable insights into the use of this enzyme.

## 2. Agar

In Japan, agar has been used as a food since several hundred years. From Japan its use extended to other oriental countries during the seventeenth and eighteenth centuries. Nowadays, agar has a wide variety of uses due to its stabilizing and gelling characteristics [11]. Agar has been mainly used in microbiological media because it is not easy for microorganisms to metabolize as well as forms clear, stable and firm gels. It is a Generally Recognized as Safe (GRAS) food additive, which is used in icings, glazes, processed cheese, jelly sweets, and marshmallows.

### 2.1. Sources of Agar

Agar is a phycocolloid extracted from the cell wall of a group of red algae (Rhodophyceae) including *Gelidium* and *Gracilaria. Gelidium* is the preferred source for agar production, but its cultivation is difficult and its natural resource is not abundant like *Gracilaria*, which is being cultivated in several countries and regions in commercial scale. Thus *Gracilaria* became an important source for agar production because it is easily harvested and cultivated [12].

### 2.2. Structures of Agar

**Figure 1:**
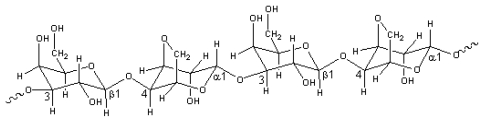
Structure of agarose.

Araki showed that agar was formed by a mixture of two polysaccharides named agarose and agaropectin [1]. The main structure of agarose is composed of repetitive units of β-d-galactose and 3,6-anhydro-α-l-galactose (3,6-AG), with few variations, and a low content of sulfate esters (Figure 1). Agaropectin has the same basic disaccharide-repeating units as agarose with some hydroxyl groups of 3,6-anhydro-α-l-galactose residues replacing by sulfoxy or methoxy and pyryvate residues [13].

Agarose has a high molecular mass above 100,000 Daltons, with a low sulfate content of below 0.15%. Agaropectin has a lower molecular mass below 20,000 Daltons, with a much higher sulfate content of 5% to 8% [14]. Agar is a mixture of agarose and agaropectin fractions in variable proportions depending on the original raw material. The concentration of agaropectin is higher in *Gracilaria*, followed by *Porphyra*, and *Gelidium* [15] (see [11] for a more detailed review of agar).

## 3. Agarases

### 3.1. Sources of Agarases

Agarases have been isolated from many sources, including seawater, marine sediments, marine algae, marine mollusks, fresh water, and soil (see Table 1 for a complete listing and references). Agarase activity has been found in seawater from Sagami Bay in Kanagawa Prefecture in Japan [6], the Bay of San Vicente in Chile [16], North Wales in UK [17], and Mediterranean Sea in France [2]. Agarase activity has been reported in extract of marine sediment collected at Ise Bay in Japan [18], at Noma Point in Japan at a depth of 230 m [19], and from the Xiamen coast in the East China Sea [20]. Because agarases are the enzymes that hydrolyzes agar, they have been isolated from the surface of rotted red algae in the South China Sea coast in Hainan Island [21], decomposing algae in Niebla in Chile [22] and in Halifax in Canada [23], and decomposing *Porphyra* in Japan [24]. Some marine mollusks live on seaweed, thus the microorganisms in their digestive tract produce carbohydrate hydrolases, such as agarases. Agarase activity has been detected in the gut of a turban shell *Turbinidae batillus cornutus* in Kangnung coast in the East Sea of Korea [25]. Most of agarases exist in the marine environment; however, some of them come from fresh water and soil. Agarases have been isolated from the IJsselmeer Lake in Netherlands [26], and soil samples from Gifu in Japan [27], Kanto area in Japan [3], and Karnataka in India [28].

Agarase activity has been reported in a wide range of microorganisms isolated from the above environments, including *Alteromonas* sp. [2,3,16,21], *Pseudomonas* sp. [23,29,30], *Vibrio* sp. [6,18,24], *Cytophaga* sp. [17,26], *Agarivorans* sp. [20,25], *Thalassomonas* sp. [19], *Pseudoalteromonas* sp. [22], *Bacillus* sp. [27], and *Acinetobacter* sp. [31], *etc.* All of the microorganisms are gram negative bacteria. Most agarases are produced extracellularly (see Table 1), except a few agarases are produced intracellularly [3,19,23,30].

### 3.2. Cleavage Pattern

**Figure 2:**
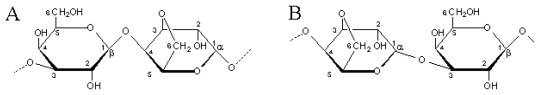
Structure of agarobiose (A) and neoagarobiose (B).

Agarases are characterized as α-agarases and β-agarases according to the cleavage pattern. The basic units of the products of α-agarases and β-agarases are agarobiose (Figure 2A) [2] and neoagarobiose (Figure 2B), respectively [3]. Identified β-agarases are more abundant than α-agarases in both the database (http://www.cazy.org/fam/acc_GH.html) and published reports. There are only two α-agarases described in the above database and literatures, *i.e.*, agarases produced by *Alteromonas agarlyticus* GJ1B from seawater [2] and *Thalassomonas* sp. JAMB-A33 from marine sediment [19].

### 3.3. Families of Agarase

Two α-agarases produced by *Thalassomonas* sp. JAMB-A33 and *Alteromonas agarlyticus* GJ1B belong to the glycoside hydrolase (GH) family 96 (http://www.cazy.org/fam/acc_GH.html). The amino acid sequences of the two α-agarases have been identified, with GeneBank accession number of BAF44076.1 (JAMB-A33) and AAF26838.1 (GJ1B), respectively. However, there is no publication available on the catalytic domain in α-agarase. The two α-agarases feature a type-VI cellulose-binding domain by the alignment of amino acid sequence in the NCBI protein database (http://www.ncbi.nlm.nih.gov/protein/6724084?report=genpept).

Amino acid sequence similarity indicates that catalytic domains of β-agarases reported up to date have been mainly classified into three GH families, *i.e.*, GH-16, GH-50, and GH-86 (http://www.cazy.org/fam/acc_GH.html). GH-16 family has most abundant members, including agarase, carrageenase, glucanase, galactosidase, laminarinase, *etc.*, while agarase is the only member of GH-50 and GH-86 families. On the other hand, most β-agarases belong to GH-16 family, while only a few β-agarases belong to GH-50 and GH-86 families (see Table 2 for a complete listing and references). Agarases from these three families carry conserved glycoside hydrolase modules that function in catalysis, and some also carry carbohydrate binding modules (CBM) [32,34].

The conserved domain of agarases in the GH-16 family has been well studied. In the characterized GH-16 enzymes, the location of GH-16 module is directly adjacent to the signal peptide, and the CBM-6 module is in the C-terminal. Such as the agarase AgaA produced by *Vibrio* sp. PO303 comprises of a typical N-terminal signal peptide of 29 amino acid residues, followed by a 266 amino acid sequence which is homologous to the catalytic module of GH-16 family, a bacterial immunoglobulin group 2 domain of 52 amino acid residues, and a 131 and a 129 amino acid sequences which are homologous to the CBM-6 family [7]. The agarase AgaV produced by *Vibrio* sp. V134 comprises of a signal peptide of 23 amino acid residues, followed by a 277 amino acid sequence of GH-16 family module, and a 152 amino acid sequence of CBM-6 family module [8]. The agarase AgaB34 produced by *Agarivorans albus* YKW-34 comprises of a signal peptide of 23 amino acid residues, followed by a 273 amino acid sequence of GH-16 family module, and a 137 amino acid sequence of CBM-6 family module [10]. As the three dimensional structure of a GH-16 family agarase has been revealed, the mechanism of catalysis and substrate binding of this family has been well understood [9,33]. The amino acid residues involving in the active site and the calcium binding site which act catalytic behavior, and those involving in the Q-X-W(F) motif and the sugar binding site which bind with the substrate are highly conserved in enzymes of this family [10].

Six β-agarases belonging to GH-50 family have been reported up to date (see Table 2 for a complete listing and references). These agarases carry partially conserved amino acid sequences of GH-50 family module extending for at least 375 amino acid residues [34]. Contrary to the GH-16 family module, the GH-50 module is located in the C-terminal end of the polypeptides.

AgrA (AAA25696) is reported to be the first agarase classified in the GH-86 family [35], however, there is no related published data. Only three agarases belonging to GH-86 family have been described in publications, *i.e.*, Aga86C and Aga86E, from *Saccharophagus degradans* 2-40 and AgaO from *Microbulbifer*-like JAMB-A94. These proteins are modular proteins consisting of CBM-6 family module and GH-86 family catalytic module [34,35]. Though the sequences are highly divergent, the critical amino acid residues (Glu, Asp) which are essential for their activity are conserved in them [35]. An interesting phenomenon is that *Saccharophagus degradans* 2-40 derived from marine algae has been reported to produce five β-agarases belonging to three different families which form a complex agarolytic system [34].

## 4. Methods for Detecting Agarase Activity

### 4.1. Qualitative Assays

Lugol’s iodine solution has been used to visualize agarase activity both for screening the agarase production by microorganism on a culture plate and for identification of protein band of agarase activity after electrophoresis [27]. It stains polysaccharide of agar into a dark brown color, while it can’t stain the degraded oligosaccharides of agar. Thus, a bright clear zone shows around the colony produce agarase and around the protein band possesses agarase activity, while other places are dark brown area.

After electrophoresis, *in situ* detection of agarase is performed on one gel, and Coomassie Brilliant Blue staining is performed on the other gel [25]. SDS in one gel is removed by rinsing the gel three times with 20 mM Tris-HCl buffer (pH 8.0) for each 10 min. Thereafter, the gel is overlaid onto a plate sheet containing 2% agar and incubated at 37 °C for 4 h. Finally, the gel is stained by Lugol’s iodine solution (5% I_2_ and 10% KI in distilled water). A clear zone is formed around the agarase band. To determine the molecular mass by electrophoresis, the other gel is stained by Coomassie Brilliant Blue after electrophoresis. The molecular mass of the agarase can be determined by comparing the two gels.

### 4.2. Quantitative Assays for Agarase Activity

Agarases degrade agar in α-1,3 or β-1,4 glycosidic bonds and from reducing ends. The agarase activity is commonly quantified by spectrophotometric determination of the increase in the concentration of reducing sugars by Nelson method [38] or DNS method [39] using d-galactose as a standard. Enzyme activity (U/mL) was defined as the amount of enzyme required to liberate 1 μmol d-galactose per min.

### 4.3. Agarase Isolation and Purification

The purification procedure of ammonium sulfate fractionation followed by anion exchange chromatography and gel filtration chromatography has often been used in purification of several agarases [3,6,18,21,22,27,31]. Ammonium sulfate fractionation may be omitted [25] or replaced by acetone precipitation [39] in this procedure. Exchangers have been used in all the above reports for capturing agarase are anion exchanger, which indicates that agarases reported are proteins with p*I* values lower than 7.

Hydroxyapatite has been successfully used for purification of a few agarases [19,35,36]. The agarase from *Thalassomonas* sp. JAMB-A33 has been purified by hydroxyapatite, followed by anion exchange chromatography, column purification by hydroxyapatite, anion exchange chromatography, and gel filtration chromatography [19]. Column purification by hydroxyapatite has been used in other cases [35,36]. Besides that, hydrophobic chromatography has been used in purification of rAgaA cloned from *Vibrio* sp. PO303 [7].

Three kinds of affinity chromatography media have been used in agarase purification up to date. The first kind of medium is the cross-linked agarose such as agarose CL-6B, which results in a marked increase of agarase specific activity based on the specific affinity of enzyme and substrate. This kind of affinity chromatography has been used cooperating with anion exchange chromatography [2,39] or gel filtration chromatography [29]. The second kind of medium is Ni^2+^ Sepharose which is commonly used to trap the 6-His tagged recombinant protein. It is used for one-step purifications of rAgaD cloned from *Vibrio* sp. PO-303 [40], five recombinant agarases cloned from *S. degradans* 2-40 [34], rAgaA from *Agarivorans* sp. LQ48 [41], and rAgaB34 cloned from *Agarivorans* sp. YKW-34 [10]. The third kind of medium is chitin bead which is used to trap intein-chitin binding domain. It is successfully applied in a one-step purification of recombinant agarase cloned from *Agarivorans* sp. JA-1 [42].

Most native and cloned agarases have been purified to high purity with a satisfactory yield. Two agarases AgaA and AgaB from the marine bacterium *Zobellia galactanivorans* have been highly purified [43], and the enzymes have been used in the growth of crystals for X-ray diffraction studies [33].

## 5. Characterization of Agarase

### 5.1. Native Agarase from Marine Environment

Various agarases from marine microorganisms derived from seawater, marine sediments, marine algae, and marine mollusks have been purified and characterized. Their important properties are listed in Table 1. Their molecular masses are highly divergent ranging from 20 to 360 kDa. The smallest agarase is produced by *Vibrio* sp. AP-2 from marine algae with a Mr of 20 kDa [24], while the largest agarase is produced by *Alteromonas agarlyticus* GJ1B from seawater with a Mr of 360 kDa [2]. As shown in Table 1, most of the agarases are composed of a single polypeptide, except the agarase produced by *Alteromonas agarlyticus* GJ1B. Using SDS-PAGE, this purified agarase has been detected as a single band with a molecular mass of 180 kDa. After the affinity-chromatography step, however, the native molecular mass was approximately 360 kDa, suggesting that the native enzyme is a dimer [2].

As shown in Table 1, the specific activities ranging from 6.3 to 292 U/mg. The reported data indicate that agarases purified from genus of *Vibrio* have lower specific activities, which are 7.54 and 20.8 U/mg from strain PO303 [18] and 6.3 U/mg from strain JT0107 [6]. Agarases from genus Agarivorans show medium specific activities, which are 57.45 and 76.8 U/mg from strain HZ105 [20] and 25.54 U/mg from strain YKW-34 [25]. Agarases from genus *Alteromonas* and *Pseudoalteromonas* exhibit high specific activities, which are 83.5 U/mg from *Alteromonas* sp. SY37-12 [21], 234 U/mg from *Alteromonas* sp. C-1 [16], and 292 U/mg from *Pseudoalteromonas antarctica* N-1 [22].

The optimal temperatures for the activity of agarases are similar. The gelling temperature of agar is around 38 °C, and most of reported agarases show optimal activity at temperature above this level (Table 1). Except agarases produced by two kinds of seawater derived bacteria, *i.e.*, *Vibrio* sp. JT0107 and *Alteromonas* sp. C-1, possess optimal activity at temperature of 30 °C [6,16], which indicates their potential application in industrial production of neoagarooligosaccharide directly from marine algae under economic conditions. Because the derivation of marine environment, most agarases are not stable in high temperature. Only agarases from *Alteromonas* sp. SY37-12 and *Agarivorans* YKW-34 have been reported to be stable up to temperature of 50 °C, others are stable up to 45 °C [2,24], 40 °C [6,19], 30 °C [16,22,23], and 25 °C [20].

Most agarases have been reported to show maximum activity at a neutral [21–23] or a week alkaline pH [2,6,17,19,25]. As we know, the natural seawater is of a week alkaline pH, thus it is reasonable that the marine derived agarase show maximum activity in this condition. Only the agarase from *Alteromonas* sp. C-1 and *Vibrio* sp. AP-2 has been reported to possess maximum activity at pH 6.5 [16] and pH 5.5 [24], respectively.

The products are totally different between different categories of the agarase. Two α-agarases derived from marine environment have been reported, *i.e.*, *Alteromonas agarlyticus* GJ1B and *Thalassomonas* sp. JAMB-A33, which produce agarotetraose as the main product. Other β-agarases have been reported to produce NA2 [23–25], NA4 [16,22] and NA6 [21] as the predominant product. Agarase from *Vibrio* sp. JT0107 has been reported to produce the mixture products of NA2 and NA4, however, NA2 is not detected when NA4 is used as a substrate [6]. Three kinds of agarases, *i.e.*, agarase-a, agarase-b, and agarase-c, secrete by a marine bacterium, *Vibrio* sp. strain PO-303, have been reported to exhibit different action patterns against agarose. Agarase-a hydrolyzed agarose into NA4 and NA6, agarase-b hydrolyzed agarose into NA2, and agarase-c produced NA6 and NA8 from agarose [18].

### 5.2. Native Agarase from Fresh Water and Terrestrial Environments

Most agarases are from marine environment, except four agarases have been reported deriving from other environments (Table 1). The molecular mass of the agarase produced by a fresh water bacteria *Cytophaga flevensis* is 26 kDa [26], while that of soil derived agarase is much higher, which is 100 kDa from *Acinetobacter* sp. AGLSL-1 [31], 113 kDa from *Bacillus* sp. MK03 [27] and 180 kDa from *Alteromonas* sp. E-l [3], respectively. The latter two kinds of agarases are reported to be dimers based on data comparison of SDS-PAGE and gel filtration. It is remarkable that the agarase from *Acinetobacter* sp. AGLSL-1 show a specific activity of 397 U/mg [31], which is the highest among the native agarases have been reported up to date. There are no special properties as comparing the above four agarases with marine derived agarases in pH and temperature properties. These four agarases are all β-agarases. It is noteworthy that agarases from *Alteromonas* sp. E-l [3] and *Acinetobacter* sp. AGLSL-1 [31] have been reported to hydrolyze agarose, NA4, and NA6 to NA2 as the only final product.

### 5.3. Cloned α-Agarase

rAgaA33 has been cloned from a deep sea sediment bacterium *Thalassomonas* sp. JAMB-A33 [44]. Two native α-agarases have been reported up to date [2,19], while rAgaA33 is the first and the only α-agarase has been reported to be recombinant produced (Table 2). It has been extracellularly produced using *Bacillus subtilis* as a host with an extraordinary production of 6950 U/L [44], whereas the native agarase has been intracellularly produced with a low production [19]. As comparing with its native agarase, its enzymatic properties including the molecular mass, the specific activity, temperature and pH activity and stability, and the final products have no significant difference with those of the native one [19,44]. Thus the recombinant α-agarase is successfully produced with a high production and maintains its native properties.

### 5.4. Cloned β-Agarases

Beside the above one α-agarase, other recombinant agarases are belonging to β-agarases. *Escherichia coli* is commonly used as a host for recombinant production of agarases (Table 2), while *Bacillus subtilis* is used for extracellular expression of recombinant β-agarases from *Microbulbifer*-like JAMB-A94, *Agarivorans* sp. JAMB-A11, *Microbulbifer thermotolerans* JAMB-A94, and *Microbulbifer* sp. JAMB-A7 [35,36,45,46]. Agarases have been intracellularly produced by *Escherichia coli* in most cases [7,34,40,42,43,47–50]. However, some agarases have been reported to be extracellularly secreted into culture media under the control of their own signal peptide in a few cases [10,39,51]. Agarases cloned from *Vibrio* sp. V134 and *Agarivorans* sp. LQ48 have been reported to exist in both culture media and cell pellets. To maintain the bioactivities, the recombinant agarases have been purified from the culture supernatant under native conditions [8,41].

A high level of extracellular production of agarase has been achieved when using *B. subtilis* as a host [35,36,45,46]. The production of recombinant agarase cloned from *Microbulbifer*-like JAMB-A94, *Agarivorans* sp. JAMB-A11, *Microbulbifer thermotolerans* JAMB-A94, and *Microbulbifer* sp. JAMB-A7 in the supernatant of the culture medium has been reported to be 7816 U/L [35], 19000 U/L [45], 19000 U/L [46], and 25831 U/L [36], which is calculated to be 80, 51, 87, and 65 mg/L, respectively. When *E. coli* has been used as a host, the production of agarase is lower than that by *B. subtilis* when calculate by mg of agarase per L of culture medium (Table 2). These reports indicate that *B. subtilis* is an efficient engineering strain which can be used for the extracellular over-expression of recombinant agarase.

The molecular mass of cloned β-agarases is ranging from 30 to 147 kDa (Table 2). Each of them has been reported to be single polypeptide with coincident molecular mass determining by SDS-PAGE and deducing from the amino acid sequence. The specific activity of them is divergent (Table 2). It is noticeable that the rAgaB cloned from a seawater derived bacterium *Pseudoalteromonas* sp. CY24 has been reported to possess a remarkable specific activity of 5,000 U/mg toward agarose [51].

As shown in Table 2, the optimal temperature for the activity of recombinant agarases is mostly around 40 °C, except those cloned from *Pseudomonas* sp. SK38 and *Agarivorans albus* YKW-34 which possess optimal activity at temperature of 30 °C [10,48] and that cloned from *Microbulbifer* sp. JAMB-A7 and *Microbulbifer thermotolerans* JAMB-A94 which exhibits optimal activity at temperature of 50 °C [36] and 55 °C [46], respectively. Most recombinant agarases are stable up to temperature of 35 °C [47,48,51], 40 °C [40,35,45] and 50 °C [10,36,41], while those cloned from *Agarivorans* sp. JA-1 and *Microbulbifer thermotolerans* JAMB-A94 are thermo-tolerant agarases which stable up to 60 °C [42,46].

As shown in Table 2, most recombinant agarases show maximum activity at a neutral or a week alkaline pH, except rAgaC cloned from *Vibrio* sp. PO-303, and rpagA cloned from *Pseudomonas* sp. SK38 possesses maximum activity at pH 6.0 [47] and pH 9.0 [48], respectively. Each recombinant agarase has a reasonable pH stability range (Table 2), except a novel agarase cloned from *Agarivorans* sp. LQ48 which is stable over a wide pH range of 3–11. Further studies by its researchers on the three-dimensional structure of this agarase will provide more information for the mechanism [41].

Agarases belonging to a same GH family display similar digestion profile toward agarose. Generally, the smallest end product or the main product is NA4 by agarases of GH-16 family, NA2 by those of GH-50 family, and NA6 or NA8 by agarases of GH-86 family (Table 2). Recombinant agarases belonging to GH-16 family are abundant, most of which have been reported to produce NA4 as the smallest end product [7,8,10,36,39,41,46], except rAgaA cloned from *Vibrio* sp. PO303 [40] and rAgaB cloned from *Zobellia galactanivorans* [43] have been reported to produce NA2 in little amount.

However, the latter two recombinant agarases cannot degrade NA4 into NA2 [40,43]. Six GH-50 family agarases have been recombinant produced, while the products of only two of them have been demonstrated, *i.e.*, agarase clone from *Agarivorans* sp. JA-1 [42] and rAgaA11 cloned from *Agarivorans* sp. JAMB-A11 [45]. These two agarases have been reported to hydrolyze not only agarose, but also NA4, to yield NA2 as the main end product [42,45].

Agarases belonging to GH-86 family are seldom found, and only two of them have been detailed described. The main product of rAgaC cloned from *Vibrio* sp. PO303 [47] and rAga86O cloned from *Microbulbifer*-like JAMB-A94 [35] is NA8 and NA6, respectively. It is noteworthy that the *agaB* gene of *Pseudoalteromonas* sp. CY24 has no significant sequence similarity with that of any known protein and the produced rAgaB hydrolyzes agarose forming NA8 and NA10 as the main end products. It has been concluded that this recombinant agarase belongs to be an unknown GH family [51].

## 6. Applications of Agarases

### 6.1. Recovery of DNA from Agarose Gel

Agarases have been widely used to recover DNA bands from the agarose gel. They are indispensable tools used in biological research field. Takara Company produces agarose gel DNA purification kit by using a β-agarase with thermo-stability up to 60 °C [52]. The agarase produced by *Vibrio* sp. JT0107 recovered 60% of the applied DNA from the gel by heating at 65 °C for 5 min [50]. Gold has reported the use of a novel gel-digesting enzyme preparation which provides an easy, rapid, and convenient method to recover PCR-amplified DNA from low melting point agarose gels [53].

### 6.2. Production of Agar-Derived Oligosaccharides

Agarases have been used for agar-derived oligosaccharides production. Comparing to the traditional acid degradation method, the enzyme degradation method has a lot of remarkable advantages, such as tender reaction condition, excellent efficiency, controllable products, simple facilities, low energy cost, little environment pollution. A simple method of preparing diverse neoagaro-oligosaccharides has been established using recombinant AgaA and AgaB cloned from *Pseudoaltermonas* sp. CY24 [51], in which agarose has been hydrolyzed into NA4, NA6, NA8, NA10, and NA12 [54].

The agar-derived oligosaccharides include agarooligosaccharides and neoagarooligosaccharides. Various reports indicated their high economic values due to their physiological and biological activities. Oligosaccharides have been prepared from agar by crude agarase from *Vibrio* QJH-12 isolated from the South China Sea coast [4]. The oligosaccharides mixture exhibit antioxidative activities in scavenging hydroxyl free radical, scavenging superoxide anion radical, and inhibiting lipid peroxidation. The oligosaccharides with the sulfate group or with higher molecular masses show stronger antioxidative activities than that without the sulfate group or with smaller molecular masses [4]. In a later report, the products mixture containing NA4 and NA6, which digested from algal polysaccharide by crude agarase products from MA103 strain, have shown high anti-oxidative properties by five *in vitro* methods [55,56]. The result indicates that neoagarooligosaccharide may have potential application in health food. Neoagarooligosaccharides have been reported to inhibit the growth of bacteria, slow down the degradation of starch, and used as low-calorie additives to improve food qualities [57]. The low polymerization degree (DP) product, such as NA2, has been reported to have a moisturizing effect on skin and a whitening effect on melanoma cells [58]. Furthermore, the higher molecular mass product, such as NA6, has been reported to exhibit as a more efficient moisturizer on skin than smaller oligosaccharides, because the viscosity of NA6 is higher than that of NA4 and NA2 [35]. Owning to these characteristics, neoagarooligosaccharides have potential applications in food, pharmaceutical, and cosmetic industries.

### 6.3. Research on Seaweed Bio-Substances and Preparation of Seaweed Protoplasts

Agarases have potential application in degrade the cell wall of red algae for extraction of labile substances with biological activities such as unsaturated fatty acids, vitamins, carotenoids from algae. Yaphe has used the agarase from *Pseudomonas atlantic* [23] in the identification of agar in marine algae (Rhodophyceae) [59]. Agarases have also been used to prepare of protoplasts. Protoplasts isolated from marine algae are useful experimental materials for physiological and cytological studies, and excellent tools for plant breeding by cell fusion and gene manipulation [60]. Araki has succeeded in the isolation of protoplasts from *Bangia atropurpurea* (Rhodophyta) by using three kinds of enzymes, *i.e.*, β-1,4-mannanase, β-1,3-xylanase, and agarase prepared from three marine bacteria, *i.e.*, *Vibrio* sp. MA-138, *Alcaligenes* sp. XY-234, and *Vibrio* sp. PO-303 [18], respectively [61]. The rAgaA, rAgaC, and rAgaD cloned from *Vibrio* sp. PO303 [7,40,47] have been reported to hydrolyze not only agarose and agar but also porphyran. Porphyran is a unique polysaccharide composing of a linear chain of alternating residues of 3-*O*-linked β-d-galactopyranose and 4-*O*-linked 3,6-anhydro-α-l-galactose. It is contained in the cell wall of *Porphyra* which is an important edible red seaweed. Hence, by combination with β-1,4-mannanase and β-1,3-xylanase, these agarases might be a useful tool for isolation of protoplasts from *Porphyra*.

## 7. Concluding Remarks

From the collective information on agarases, we conclude that agarases are important enzymes naturally purified and cloned from variety of microorganisms. They are characterized into two categories with more than four families which can produce various oligosaccharides with different DP values. A few agarases have a remarkable production and possess excellent properties such as high specific activity, excellent temperature and pH stability, which enlighten their widely potential applications.

## Figures and Tables

**Table 1:** Agarase from marine bacteria: localization, characteristics, and products.

Source	Localization	Category (α/β agarase)	Mr(kDa)	Specific activity (U/mg)	Optimal T (°C)	Stable up to T (°C)	Optimal pH	Stable pH	Product	Ref.

**Sea water**										
*Vibrio* sp. JT0107	extracellular	β	107	6.3	30	40	8.0	-	NA2, NA4	[6]
*Alteromonas* sp. C-1	extracellular	β	52	234	30	30	6.5	-	NA4	[16]
*Cytophaga* sp.	extracellular	β	-	-	40	-	7.2	-	-	[17]
*Alteromonas agarlyticus* GJ1B	extracellular	α	180 (SDS-PAGE) 360 (gel filtration)	-	-	45	7.2	>6.5	A4	[2]
**Marine sediment**										
*Vibrio* sp. PO-303	extracellular	β	87.5 115 57	7.54 28.4 20.8	38–55	-	6.5–7.5	-	NA4, NA6 NA2 -	[18]
*Thalassomonas* sp. JAMB-A33	intracellular	α	85	40.7	45	40	8.5	6–11	A2, A4 (main), A6	[19]
*Agarivorans* sp. HZ105	extracellular	β	58 54	76.8 57.45	-	25	6.0–9.0	-	-	[20]
**Marine algae**										
*Alteromonas* sp. SY37-12	extracellular	β	39.5	83.5	35	50	7.0	-	NA4, NA6 (main), NA8	[21]
*Pseudoalteromonas antarctica* N-1	extracellular	β	33	292	-	30	7	-	NA4, NA6	[22]
*Pseudomonas atlantica*	intracellular	β	32	-	-	30	7.0	6.5–7.5	NA2	[23]
*Vibrio* sp. AP-2	extracellular	β	20	-	-	45	5.5	4.0–9.0	NA2	[24]
**Marine mollusks**										
*Agarivorans albus* YKW-34	extracellular	β	50	25.54	40	50	8.0	6.0–9.0	NA2 (main), NA4	[25]
**Fresh water**										
*Cytophaga flevensis*	extracellular	β	26	-	35	40	6.3	6.0–9.0	NA2, NA4, NA6…NA16	[26]
**Soil**										
*Bacillus* sp. MK03	extracellular	β	92 (SDS-PAGE) 113 (gel filtration)	14.2	40	35	7.6	7.1–8.2	NA2, NA4(main)	[27]
*Alteromonas* sp. E-l	intracellular	β	82 (SDS-PAGE) 180(gel filtration)	34	40	40	7.5	7–9	NA2	[3]
*Acinetobacter* sp. AGLSL-1	extracellular	β	100	397	40	45	6.0	5.0–9.0	NA2	[31]
**Unknown**										
*Pseudomonas*-like bacteria	extracellular	β	210 63	-	38 43	- -	6.7 6.7	- -	NA2, NA4, NA6 NA4	[29]
*Pseudomonas atlantica*	intracellular	β	-	-	-	-	-	-	NA4, NA6, NA8, NA10	[30]

**Table 2:** Recombinant agarases from engineered microorganisms: localization, characteristics, products, and accession number.

Source	Expression strain	Name of the gene	Localization	Production		GH Family	Mr (kDa)	Specific activity (U/mg)	Optimal T (°C)	Stable up to T (°C)	Optimal pH	Stable pH	Product	GeneBank accession number	Ref.
				(U/L)	(mg/L)										
**β-agarase**															
*Pseudomonas* sp. W7	*Escherichia coli* JM83	-	extracellular	-		16	59	-	20–40	-	7.8	-	NA4	AAF82611	[39]
*Vibrio* sp. PO-303	*Escherichia coli* BL21	*agaA*	intracellular	-		16	106	16.4	40	-	7.5	-	NA4, NA6	BAF62129	[7]
*Vibrio* sp. PO-303	*Escherichia coli* DH5α	*agaD*	intracellular	620	10	16	51	63.6	40	45	7.5	4–9	NA4(main), NA2, NA6	BAF34350	[40]
*Vibrio* sp. PO-303	*Escherichia coli* BL21	*agaC*	intracellular	7130	22	86	51	329	35	37	6.0	4–8	NA4, NA6, NA8(main)	BAF03590	[47]
*Agarivorans* sp. JA-1	*Escherichia coli* DH5α	-	intracellular	554	3	50	109	167	40	60 (70%)	8.0	-	NA2, NA4	ABK97391	[42]
*Saccharophagus degradans* 2–40	*Escherichia coli* EPI300	*aga50A*	intracellular	-		50	87	-	-	-	-	-	-	ABD80438	[34]
		*aga16B*				16	64							ABD80437	
		*aga86C*				86	86							ABD81910	
		*Aga50D*				50	89							ABD81904	
		*aga86E*				86	146							ABD81915	
*Microbulbifer*-like JAMB-A94	*Bacillus subtilis*	*agaO*	extracellular	7816	80	86	127	98	45	40	7.5	6–9	NA6 (main)	BAD86832	[35]
*Zobellia galactanivorans*	*Escherichia coli* DH5α	*agaA*	intracellular	160	1	16	60	160	-	-	-	-	NA4, NA6	AAF21820	[43]
		*agaB*		800	8		31	100					NA2, NA4	AAF21821	
*Agarivorans* sp. JAMB-A11	*Bacillus subtilis*	*agaA11*	extracellular	19000	51	50	105	371	40	40	7.5–8.0	6–11	NA2 (main)	BAD99519	[45]
*Microbulbifer thermotolerans* JAMB-A94	*Bacillus subtilis*	*agaA*	extracellular	45000	87	16	48	517	55	60	7.0	6–9	NA4 (main)	BAD29947	[46]
*Microbulbifer* sp. JAMB-A7	*Bacillus subtilis*	*agaA7*	extracellular	25831	65	16	49	398	50	50	7.0	5–8	NA4	BAC99022	[36]
*Pseudomonas* sp. SK38	*Escherichia coli* BL21	*pagA*	intracellular	-		16	37	32.3	30	37	9.0	8–9	-	AF534639	[48]
*Vibrio* sp. JT0107	*Escherichia coli* DH5α	*agaA*	intracellular	-		50	105	-	-	-	-	-	-	BAA03541	[50]
		*agaB*		-		50	103							BAA04744	[49]
*Vibrio* sp. V134	*Escherichia coli* BL21	*agaV*	extracellular/intracellular	-		16	52	-	40	-	7.0	-	NA4, NA6	ABL06969	[8]
*Agarivorans albus* YKW-34	*Escherichia coli* DH5α	*agaB34*	extracellular	1670	7	16	30	242	30	50	7.0	5–9	NA4 (main)	ABW77762	[10]
*Agarivorans* sp. LQ48	*Escherichia coli* BL21	*agaA*	extracellular/intracellular	-		16	51	349.3	40	50	7.0	3–11	NA4, NA6	ACM50513	[41]
*Pseudoalteromonas* sp. CY24	*Escherichia coli* BL21	*agaB*	extracellular	17000	3	Novel	51	5000	40	35	6.0	5.7–10.6	NA8, NA10	-	[51]
**α-agarase**															
*Thalassomonas* sp. JAMB-A33	*Bacillus subtilis*	*agaA33*	extracellular	6950	155	96	87	44.7	45	-	8.5	6.5–10.5	NA4 (main)	BAF44076.1	[44]
